# Atenolol is associated with salivary gland dysfunction and sialochemical alterations, along with redox imbalance and structural changes in male rats

**DOI:** 10.1007/s00210-026-05430-6

**Published:** 2026-05-16

**Authors:** Helder Carlos Pelais Freitas, Cristian Kallahan Silva Chagas, Wallacy Watson Pereira Melo, Cristian dos Santos Pereira, Hannah Gil de Farias Morais, Roseana de Almeida Freitas, Renata Duarte Souza-Rodrigues, Antonio Hernandes Chaves-Neto, Rafael Rodrigues Lima

**Affiliations:** 1 Laboratory of Functional and Structural Biology, Institute of Biological Sciences, Federal University of Pará, Rua Augusto Corrêa, nº 01, Guamá, Belém, Pará, 66075-110 Brazil; 2 Multicampi School of Medical Sciences, Federal University of Rio Grande Do Norte, Caicó, Brazil; 3 Department of Basic Sciences, School of Dentistry, São Paulo State University (UNESP), Araçatuba, Brazil

**Keywords:** Dentistry, Antihypertensive drugs, Atenolol, Toxicology, Salivary glands

## Abstract

This study aimed to evaluate the effects of atenolol administration on salivary gland structure, redox status, and sialochemical composition in male rats. Male Wistar rats (Rattus norvegicus), aged 90 days, were randomly assigned to two groups: a control group receiving distilled water and an atenolol-treated group receiving 20 mg/kg/day by orogastric gavage for 30 days. On day 31, the animals were anesthetized, saliva was collected, and the parotid, submandibular, and sublingual glands were harvested for oxidative, morphometric, histopathological, and biochemical analyses. The results showed no systemic changes in plasma parameters. However, atenolol administration was associated with local redox imbalance in salivary glands, accompanied by alterations in salivary biochemistry, including reduced antioxidant capacity and increased pro-oxidant markers (lipid peroxidation and protein carbonylation). Morphological analysis revealed acinar atrophy in all glands, as well as reduced stroma and collagen fibers in the parotid and submandibular glands. Sialochemical analysis demonstrated decreased levels of total protein, mucin, and phosphate, along with increased calcium concentration. Atenolol use was associated with sialochemical disturbances and structural alterations in salivary glands, accompanied by inflammatory cell presence and changes in oxidative stress markers in both tissue and saliva, suggesting potential implications for oral health.

## Introduction

Systemic arterial hypertension affects approximately 1.4 billion adults aged 30–79 years worldwide, representing a major global public health challenge characterized by persistently elevated blood pressure (Dodulík et al. 2025; WHO 2025). In response to this burden, several pharmacological classes have been developed to control blood pressure and improve patient outcomes (Gao et al. 2021).

Among these, beta-blockers remain widely prescribed, with atenolol being one of the most commonly used agents (Mengue et al. 2016; Lee and Chun 2024). Atenolol selectively antagonizes β₁-adrenergic receptors in cardiac tissue, reducing the inotropic and chronotropic effects of endogenous catecholamines and, consequently, sympathetic activity (Liang and Zhou 2022). However, despite its cardioselectivity, systemic adrenergic blockade may also affect non-target tissues that depend on sympathetic stimulation, including the pulmonary system (Tiotiu et al. 2019), peripheral circulation (Paravastu et al. 2013), and the oral cavity (Liang and Zhou 2022).

In the oral cavity, beta-blocker use has been associated with alterations in salivary flow and composition, potentially compromising lubrication, dental remineralization, and antimicrobial defense (Lima et al. 2018; Salvatori et al. 2016). These effects may be related to the heterogeneous distribution of β-adrenergic receptors in the major salivary glands. The parotid gland, for instance, exhibits a higher density of β₁ receptors, which may increase its susceptibility to atenolol and impair the secretion of protein-rich serous saliva (Ryberg et al. 2008; Proctor and Carpenter 2014).

Despite the widespread use of beta-blockers, the biochemical and morphological consequences of chronic atenolol exposure on salivary glands remain incompletely understood (Varellis et al. 2020). Although reductions in salivary flow have been reported, there is still limited evidence regarding associated mechanisms, including histopathological alterations and oxidative stress (Anwar et al. 2022; Ramírez et al. 2023). Experimental studies suggest that prolonged adrenergic modulation may influence protein expression and redox balance in salivary glands, affecting the parotid, submandibular, and sublingual tissues (van Kuijk et al. 2010; Navajas et al. 2014).

Therefore, this study aimed to evaluate the effects of chronic atenolol administration on the major salivary glands of rats, hypothesizing that its use is associated with structural, sialochemical, and redox alterations.

## Materials and methods

### Experimental groups

**Fig. 1:**
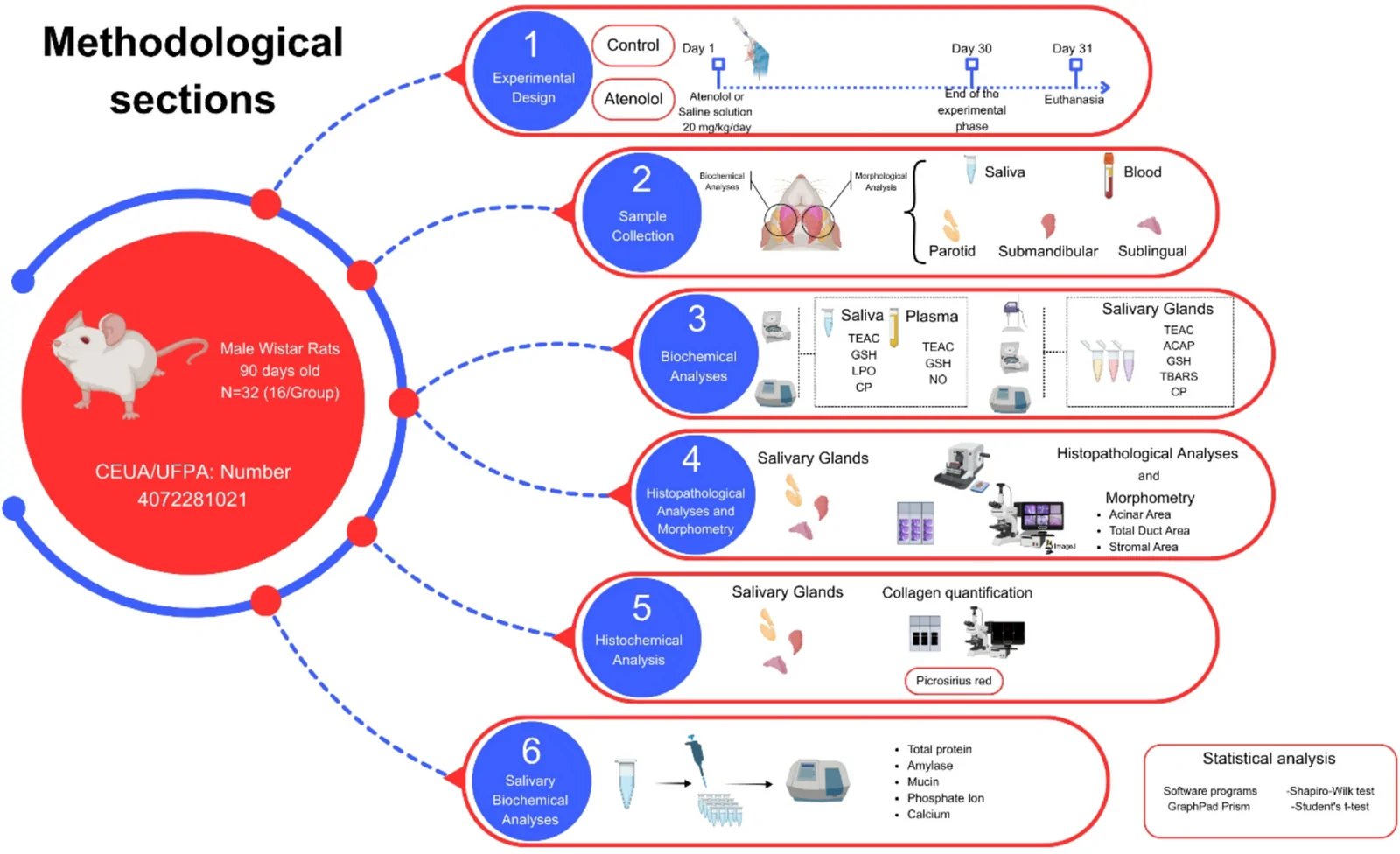
Methodological summary of atenolol administration. Collection of salivary glands, blood, saliva, and analyses. (1) Experimental design (2) Sample collection (3) Biochemical analyses (4) Histopathological and morphometric analyses (5) Histochemical analysis (6) Salivary functional analysis

The experimental procedures, as shown in Fig. 1, were approved by the Animal Experimentation Ethics Committee of the Federal University of Pará (CEUA-UFPA N° 4072281021) and followed the recommendations of the National Institutes of Health Guide for the Care and Use of Laboratory Animals: Report on In Vivo Experiments (Institute of Laboratory Animal Resources 2011).

The study used 32 male albino rats (*Rattus norvegicus*, Wistar strain), approximately 90 days old and weighing about 250 g, which were kept under controlled environmental conditions (temperature: 25 ± 1 °C; relative humidity: 40–70%; 12/12 h light/dark cycle). The animals were housed in polypropylene cages, four per unit, with free access to water and a balanced diet (Labina, Supralab®, São Leopoldo, RS, Brasil). The sample size was calculated using GPower software, using the t-test family, adopting total protein concentration as the primary outcome with an effect size of 0.9, a 95% confidence interval (alpha error), 80% power (beta error), and an allocation ratio of 1, providing a sufficient *n* = 32. Using an online tool (http://graphpad.com/quickcalcs/randomise1.cfm), the rats were randomly divided into two groups: the control group (*n* = 16), which received distilled water at the same rate of administration as the drug, and the atenolol group (*n* = 16), which received the drug at a dose of 20 mg/kg/day.

### Atenolol administration

Atenolol (Sandoz, Holzkirchen, Germany) was administered by orogastric gavage once a day at a fixed time for 30 consecutive days (Zhang et al. 2004). The dose administered was 20 mg/kg/day, based on previous studies on the antihypertensive and cardioprotective effects of atenolol (Shen et al. 2005). This dose is safe and does not cause acute toxicity (Xie et al. 2002; Xu et al. 2004). The animals in the control group received distilled water for the same period, in a volume similar to that of the drug. All animals were weighed every 7 days to adjust the dosage.

### Sample collection and processing

On the 31st day of the experiment, the animals, already 120 days old, were anesthetized with a mixture of ketamine hydrochloride (90 mg/kg) and xylazine hydrochloride (9 mg/kg), and upon the loss of corneal reflexes, they received an intraperitoneal injection of pilocarpine (1 mg/kg, pilocarpine hydrochloride, Sigma-Aldrich, USA) to allow saliva collection in plastic microtubes cooled with ice for 10 min. The saliva samples were centrifuged (Modelo K14-0815, Kasvi, São José dos Pinhais, PR, Brasil) at 3000 rotations per minute (rpm) for 10 min, and the supernatant was immediately stored in dry ice and later in a -80 °C freezer (de Moraes Ramos et al. 2006; Nascimento et al. 2021). Subsequently, euthanasia was performed by exsanguination, and blood was collected with a syringe and stored in vacuum collection tubes with Ethylenediaminetetraacetic acid (EDTA). Blood samples were centrifuged at 3000 rpm for 10 min, and plasma was stored at -80 °C.

After blood collection, salivary glands (parotid, submandibular, and sublingual) were surgically excised for biochemical analyses, immediately frozen in dry ice, and stored at − 80 °C (Dos Santos et al. 2022). After thawing, the samples were homogenized by ultrasonic disruption in 20 mmol/L Tris–HCl buffer (pH 7.4) at 4 °C (approximately 1 g/mL), followed by centrifugation at 3000 rpm for 10 min. The supernatant was collected and stored at − 80 °C until analysis.

Following surgical removal of glands for biochemical assays, the animals were perfused through the left ventricle with 0.9% heparinized saline, followed by 4% formaldehyde. Subsequently, additional salivary glands were collected for morphological evaluation and fixed in 4% neutral buffered formalin for 24 h prior to histological and histochemical analyses.

### Oxidative biochemical analysis

Plasma samples, homogenized salivary glands, and saliva were subjected to analysis of antioxidant and pro-oxidant parameters, with the proteins in the samples quantified for correction of biochemical data (Bradford 1976).

#### Measurement of antioxidant capacity equivalent to trolox (TEAC)

This analysis was performed on plasma, salivary gland, and saliva samples. The technique used to measure Trolox equivalent antioxidant capacity (6-hydroxy-2,5,7,8-tetramethylchromium-2- carboxylic acid; Sigma-Aldrich 23,881–3) was employed. For this purpose, the method proposed by Miller et al. (1993), modified by Re et al. (1999), was used. This colorimetric technique is based on the reaction between 2,2-azinobis[3-ethylbenzothiazoline-6-sulfonate] diammonium salt (ABTS; Sigma-Aldrich A1888) and potassium persulfate (K_2_S_2_O_8_; Sigma-Aldrich 60,490), which produces the 2,2-azinobis[3-ethylbenzothiazoline-6-sulfonate] diammonium salt radical (ABTS^+^•), a green/blue chromophore. This analysis of salivary glands and saliva has already been established in previous studies (Lima-da-Silva et al. 2025) and blood (Kubota et al. 2026), with results expressed in µmol/g of protein.

#### Antioxidant capacity against peroxyl radicals (ACAP)

This test evaluates the response of parotid, submandibular, and sublingual glandular tissue samples to a reagent that generates reactive oxygen species (ROS), according to the protocol established by Amado et al. (2009) and used in previous studies (Lima-da-Silva et al. 2025). For this purpose, 2,2’-azobis(2-methylpropionamidine) dihydrochloride (AAPH; 4 mM; Sigma-Aldrich, St. Louis, MI, USA) was thermally decomposed at 35 °C. Aliquots of the samples were distributed into two microplates for analysis, one containing Milli-Q water and the other supplemented with the AAPH reagent. The procedure for evaluating antioxidant capacity detects ROS concentrations by measuring the fluorescence emitted by 2,7-Dichlorofluorescin (H_2_DCF-DA, Invitrogen™, Waltham, MA, USA) at a final concentration of 40 nM. Increased fluorescence indicates higher levels of ROS in the samples. Therefore, the samples were examined in the presence and absence of AAPH (a peroxyl radical generator) to quantify peroxyl radicals under both conditions (Ferreira et al. 2021).

Fluorescence readings of samples with and without AAPH were taken every five minutes for one hour using a fluorescence microplate reader (Victor X3; Perkin Elmer, Waltham, MA, USA). Antioxidant capacity was determined by comparing the relative differences in ROS levels between the two conditions. Since the method measures unextinguished free radicals, which are inversely related to antioxidant capacity, the data were presented as the inverse of the relative area, effectively representing antioxidant activity.

The principle underlying the method are that samples with higher antioxidant activity neutralize more peroxyl radicals generated by AAPH, leading to reduced fluorescence emissions. Total fluorescence was determined by integrating the fluorescence units (FU) over the duration of the readings and fitting the data to a second-order polynomial equation. The results were expressed as the difference in FU/min area between AAPH -treated and untreated samples, normalized by the area of the untreated sample. The inverse of this relative difference served as a measure of antioxidant capacity. To ensure accurate results, protein concentrations were analyzed to standardize sample dilutions prior to testing.

#### Glutathione reduced (GSH) assay

Glutathione was determined according to the methodology adapted by Ellman (1959). An aliquot of 20 μL of the samples was mixed in a tube containing 20 μL of distilled water and 3 mL of Phosphate-Buffered Saline/EDTA solution (pH 8.0) for the first measurement. Subsequently, 5,5’-dithiobis (2- nitrobenzoic) acid (DTNB; 0.47 mmol) was added to the solution, and a new measurement was performed after 3 min. This analysis was performed on plasma, salivary gland, and saliva samples, and the results obtained were normalized by total protein levels (Matos-Sousa et al. 2025). The results were expressed in mg of GSH/mg of protein.

#### Measurement of thiobarbituric acid reactive substances (TBARS)

After filling with 0.5 mL of 10 nM Thiobarbituric Acid (TBA) reagent and 250 μL for samples from the submandibular, parotid, and sublingual glands, each tube was placed in a water bath at 94 °C for 1 h, cooled to room temperature for 15 min, and then received 4 mL of butyl alcohol. The samples were then homogenized in a vortex to extract the products of polyunsaturated lipid peroxidation, such as malondialdehyde (MDA), a byproduct of fatty acid degradation in the cell membrane. The tubes were centrifuged at 2,500 rpm for 10 min, and the MDA concentration (nmol/g of protein) was determined by spectrophotometry at 535 nm using the method described by Kohn and Liversedge (1944), as adapted by Percário et al. (1991).

#### Lipid peroxidation (LPO) assay

The level of lipid peroxidation in saliva was determined using the method proposed by Esterbauer and Cheeseman (1990), based on the reaction of polyunsaturated fatty acid metabolites, MDA and 4-hydroxyalkenes (4-HA), with N-methyl-2-phenylindole (NMFI). This reaction, in the presence of methanesulfonic acid, produces a stable chromophore whose color is proportional to the concentration of oxidized lipids and can be measured spectrophotometrically. After centrifugation at 3500 rpm for 10 min at 4 °C, the supernatants and MDA standard solutions were incubated with a 1:4 solution of methanesulfonic acid and 10.3 mM N-methyl-2-phenylindole diluted in methanol (1:3) at 45 °C for 40 min, followed by spectrophotometric reading (λ = 570 nm). After correction for protein concentration, lipid peroxidation results were expressed in nmol/g of protein.

#### Nitric oxide (NOx) levels

The concentration of NOx in plasma was determined indirectly by detecting NO₃⁻ (nitrate) or NO₂⁻ (nitrite) in the samples using the Nitric Oxide colorimetric assay kit (Elabscience®, Catalog No.: E-BC- K035-M). Initially, 100 µL of the standard was mixed with 200 µL of reagent 1 (sulfate solution) and 100 µL of reagent 2 (alkaline reagent). After 15 min of rest, the solution was centrifuged at 3000 rpm for 10 min. Next, 160 µL of the supernatant was transferred to a microplate, where 80 µL of the chromogenic reagent was added. After 15 min of incubation at room temperature, spectrophotometric readings were taken at 550 nm (Green et al. 1981). The results were expressed in µmol/g of protein after correction.

#### Measurement of carbonyl protein (CP)

This measurement was performed on samples from the parotid, submandibular, sublingual glands, and saliva, following the protocol (Mesquita et al. 2014). To prepare the solutions used in the analyses, a 0.5 mol/L phosphoric acid solution was initially prepared by diluting a 1 mol/L phosphoric acid solution (Sigma Aldrich, code: 329,761,708). For this, equal volumes of deionized water and the 1 mol/L phosphoric acid solution were homogenized. Next, a 10 mmol/L 2,4-dinitrophenylhydrazine (DNPH; Sigma Aldrich; molar mass 198.14 g/mol) solution was prepared using solid DNPH dissolved in a 0.5 mol/L phosphoric acid solution. For a final volume of 50 mL, 0.0991 g of DNPH was weighed out and initially mixed with 35 mL of the acid solution under heated magnetic stirring, using a glass rod to disperse any lumps. As the DNPH did not dissolve completely, the solution was cooled, distributed into 15 mL conical tubes, and centrifuged briefly to allow the particulate material to settle. The supernatant was then transferred to a 50 mL volumetric flask and the volume was made up with 0.5 mol/L phosphoric acid solution. The final solution was stored in an amber glass bottle and kept refrigerated. Next, a 6 mol/L sodium hydroxide (NaOH) solution (25 mL) was prepared by diluting 6 g of NaOH in deionized water, allowing it to cool before completing the volume in the volumetric flask. This solution was stored in a plastic bottle.

For the analysis, 100 μL of the sample or 50 mmol/L potassium phosphate buffer (blank) was added. Next, 100 μL of DNPH solution was added to each well, and incubation was performed for 10 min at room temperature and protected from light. After this period, 50 μL of NaOH solution was added, ensuring vigorous shaking to homogenize the reagents in each well. The reaction was incubated again for 10 min under the same temperature conditions and protected from light. Finally, the absorbance was measured spectrophotometrically at 450 nm. The results were expressed in nmol/mg of protein.

### Tissue analysis of the salivary glands

Samples from the parotid, submandibular, and sublingual glands were immersed in 4% formalin for 24 h for fixation. They were then dehydrated in ethanol solutions of increasing concentrations (70%, 80%, 90%, absolute 1, and absolute 2), clarified in xylene, and then embedded in paraffin for 30 min. The samples were once again embedded in paraffin again for 30 min and fixed in Leuckart blocks. The blocks were sectioned using a manual microtome (YIDI Medical Appliance Factory YD-315 Rotary Microtome), adjusted for 5 µm thick sections for histopathological, morphometric, and histochemical analyses.

#### Histopathological analysis

For this analysis, slides with samples from the parotid, submandibular, and sublingual glands, stained with hematoxylin and eosin (H&E), were examined to identify possible pathological changes in the glandular tissue, lining epithelium, and connective tissue, considering the secretory units, the ductal system, and the surrounding stroma. Representative photomicrographs were obtained using a color digital camera (Eclipse C, DS-Fi3, Nikon, Tokyo, Japan) attached to a microscope (Eclipse Ci-S, Nikon, Tokyo, Japan; 40 × magnification). Two pathologists, without prior knowledge of the experimental data, analyzed the slides qualitatively and independently using a microscope (Eclipse E200, Nikon, Tokyo, Japan; 40 × magnification). Cohen’s kappa coefficient was applied to assess interobserver agreement, resulting in excellent agreement (κ = 0.93) (Melo et al. 2026).

#### Morphometric analysis

Sections stained with hematoxylin and eosin were obtained for morphometric analysis considering the area of acini, ducts, and stroma, following the protocol adapted from (Ferreira et al. 2021). Three photomicrographs per animal were obtained for this analysis using a color digital camera (Eclipse C, DS-Fi3, Nikon, Tokyo, Japan) attached to a microscope (Eclipse Ci-L, Nikon, Tokyo, Japan, 40 × magnification). Each photomicrograph was analyzed using the Color Threshold function of Fiji ImageJ software version 2.4, selecting the region of interest (ROI) for all parameters analyzed.

After defining the scale of the photomicrographs, brightness adjustments in the color threshold function delimited the parenchyma of the salivary gland (acini and ducts) and the total stromal area. To measure the ducts, the freehand selection tool was used to measure each duct in the image. The sum of the area of each duct was obtained and subtracted from the total parenchymal area previously measured, resulting in the total area of the acini shown in the same image. The results of the three photomicrographs were averaged for each parameter. The results obtained were expressed in μm^2^ and converted to mm^2^. All measurements are described in mm^2^.

#### Histochemical analysis

To evaluate the collagen content of the salivary glands, the slides were stained with Picrosirius Red and counterstained with Harris hematoxylin (Souza-Monteiro et al. 2025). Three sections of each sample were then examined under a polarized light microscope (Eclipse Ci-S, Nikon, Tokyo, Japan, 40 × magnification), and three photomicrographs of each region were obtained using a color digital camera (Eclipse Ci-S, Nikon, Tokyo, Japan; 40 × magnification) attached to the microscope. The thresholding tool of the ImageJ software (NIMH, NIH, Bethesda, MD, USA, http://rsbweb.nih.gov/ij/) was used to delimit the collagen area, using the arithmetic mean of the sections to determine the collagen area of the sample, which was measured in µm^2^ and transformed to mm^2^.

### Salivary biochemical analyses

In order to evaluate a functional profile of saliva, in addition to the oxidative biochemical analyses described above, total protein levels, amylase activity, mucin levels, and calcium and phosphorus ion quantities were evaluated.

#### Salivary protein analysis

Total protein concentration was determined according to the protocol proposed by Bradford (1976), in which proteins were bound to Coomassie Brilliant Blue dye to form a blue compound with maximum absorbance at 595 nm, using a spectrophotometer. The data obtained were tabulated and compared with the reference values stipulated by Giacomello et al. (2020). The results were expressed in g/dL.

Amylase activity was determined according to the method described by Caraway (1959) and modified by Rinderknecht et al. (1967) and Searcy et al. (1967), in which colorimetric detection was performed (K003 BIOCLIN, Quibasa, Belo Horizonte, Minas Gerais, Brazil). Absorbance was measured spectrophotometrically at 660 nm. Results were expressed in U/dL.

Mucin was analyzed using a kit for detecting protein concentration (Bioclin, Belo Horizonte, Minas Gerais, Brazil). The results were expressed in mg/dL.

#### Saliva ion analysis

The parameters were determined using commercial colorimetric kits from Bioclin®, strictly following the manufacturer’s instructions. Calcium was determined using the Arsenazo III Calcium kit (K051-2), in which calcium forms a stable complex with the Arsenazo III dye, with absorbance recorded at 680 nm. The results were expressed in mg/dL.

Phosphate analysis was performed using the phosphorus kit (K020-1). This analysis is based on the reaction of the phosphate ion present in the sample with ammonium molybdate in an acidic medium, forming the phosphomolybdate complex. This complex, in the presence of a reducing agent provided by the kit reagent, produces a colored product whose intensity is directly proportional to the phosphorus concentration, with a reading at 650 nm. The results were expressed in mg/dL.

### Statistical analysis

Statistical analyses were performed using GraphPad Prism 7.0 software (GraphPad Software, San Diego, California). The data obtained from the experimental groups were tabulated, and normality was tested using the Shapiro–Wilk method (*p* < 0.05). The data were analyzed using Student’s t-test, with a significance level of *p* < 0.05.

## Results

### In a systemic setting, the drug atenolol did not cause changes in the redox state in blood plasma

**Fig. 2:**
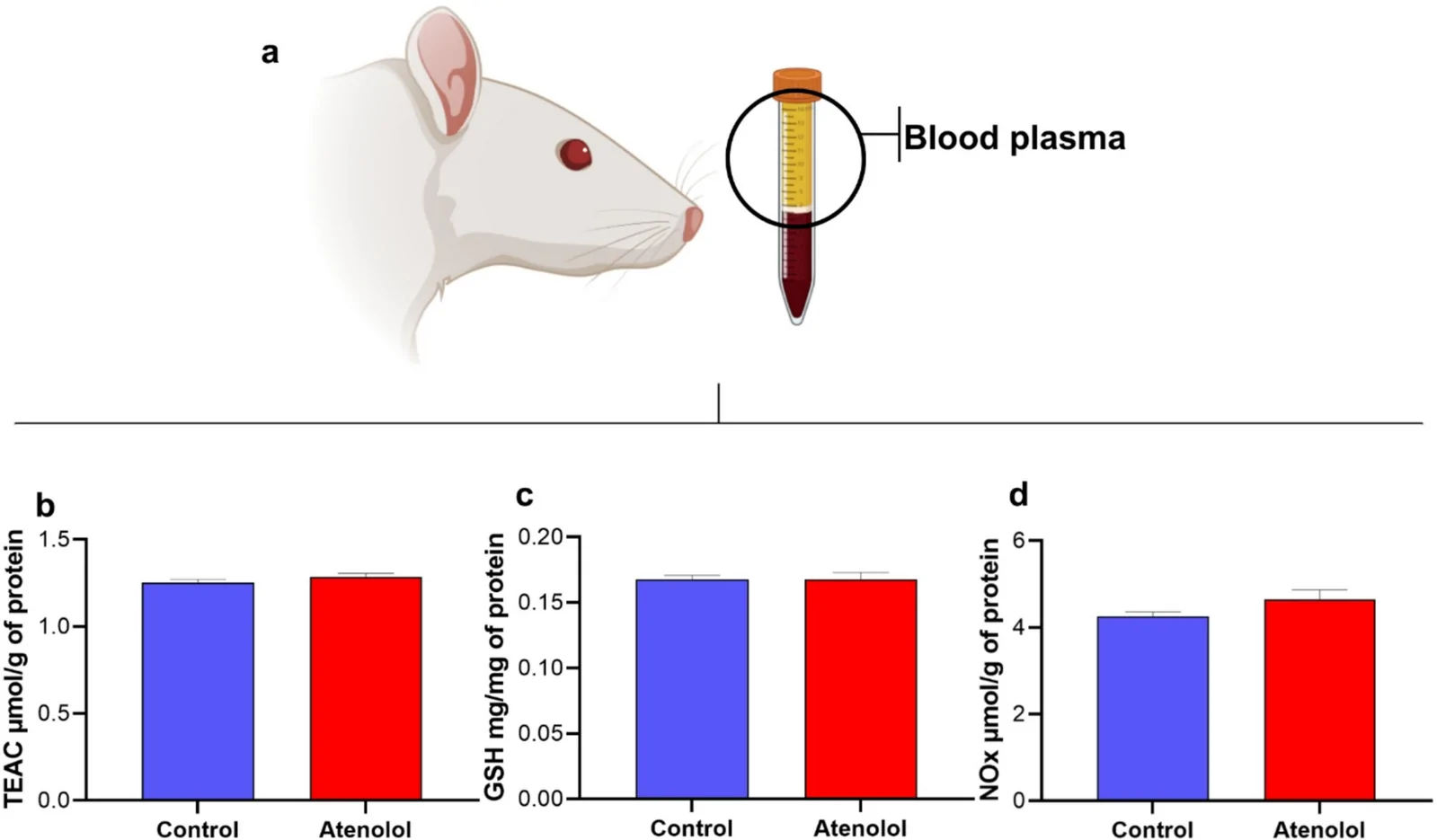
Effect of atenolol administration (20 mg/kg/day) on blood redox state. In **A**, representation of the sample used. In **B**, antioxidant capacity equivalent to Trolox (TEAC; µmol/g of protein); in **C**, glutathione levels (GSH; mg/mg of protein); in **D**, nitric oxide metabolites (NOx; µmol/g of protein). Quantitative results are expressed as mean ± standard error of the mean (SEM), Student’s t-test (*n* = 16). * Student’s t-test, *p* < 0.05

Blood plasma (Fig. 2) data showed that there were no changes in the redox state when analyzing the antioxidant parameters TEAC (Control: 1.25 ± 0.01; Atenolol: 1.28 ± 0.02; *p* = 0.25) and GSH (Control: 1.16 ± 0.003; Atenolol: 1.16 ± 0.005; p > 0.99), as well as the pro-oxidant parameter NOx (Control: 4.25 ± 0.10 µM/L; Atenolol: 4.64 ± 0.22; *p* = 0.14).

### However, atenolol promoted a reduction in antioxidant capacity associated with an increase in pro-oxidant activity in the salivary glands

**Fig. 3:**
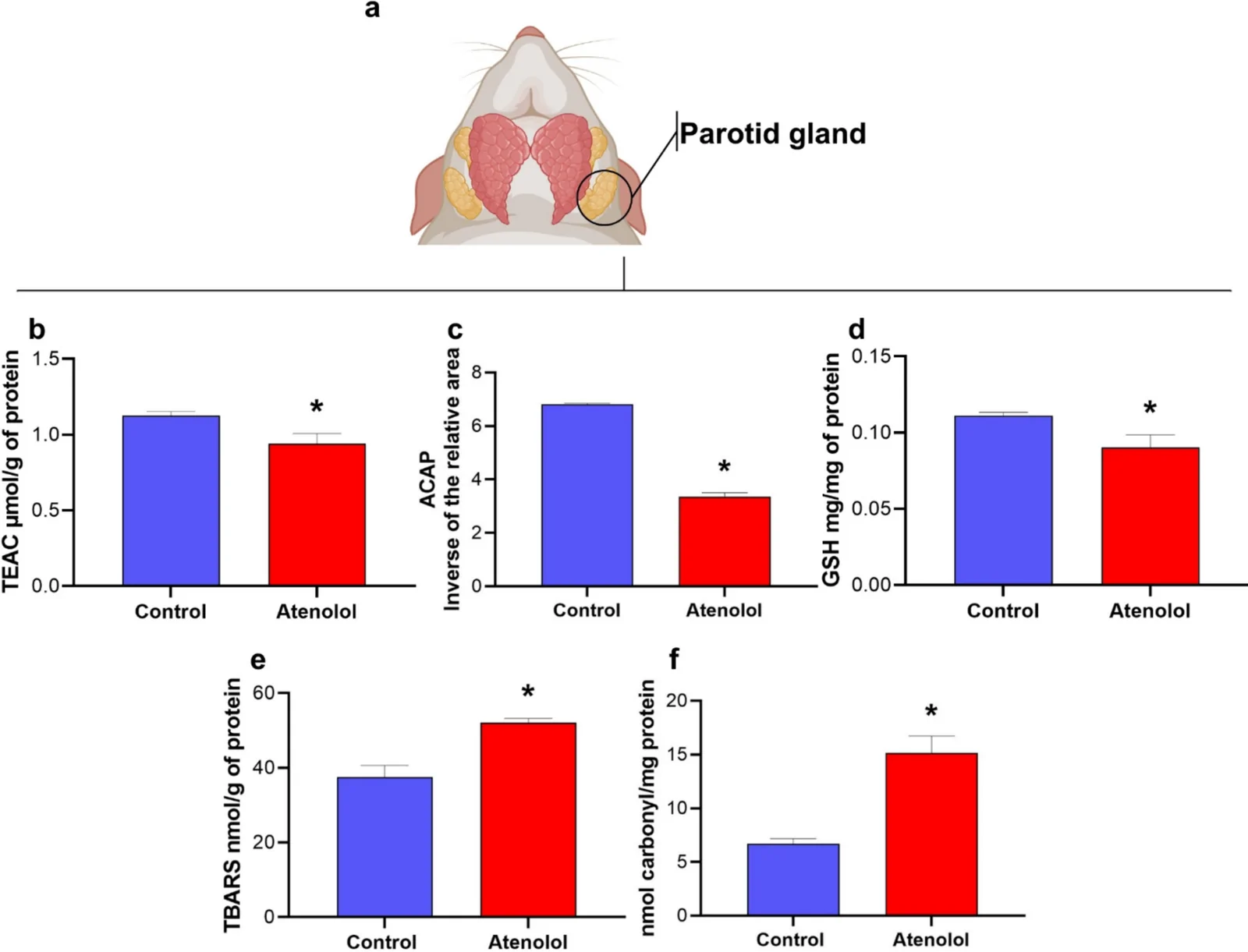
Effect of atenolol on the redox state of the parotid gland. **A** Schematic representation of the anatomical location of the parotid gland in rats. Graphical representation of the analysis of antioxidant parameter data (**B**) TEAC—antioxidant capacity equivalent to Trolox; **C** ACAP—Antioxidant capacity against peroxyl radicals; **D** GSH—glutathione levels. Graphical representation of the analysis of pro-oxidant parameter data (**E**) TBARS—thiobarbituric acid reactive substances (**F**) CP – Carbonyl Proteins. Oxidative biochemistry expressed as mean ± SEM, Student’s t-test, * *p* < 0.05

Unlike the blood analysis, the parotid gland data showed a reduction in both antioxidant capacity and glutathione levels in the atenolol group compared to the control group (Fig. 3). In the TEAC analysis (Control: 1.12 ± 0.02; Atenolol: 0.94 ± 0.06; *p* = 0.03), ACAP (Control: 6.81 ± 0.026; Atenolol: 3.35 ± 0.14; *p* < 0.0001) and GSH (Control: 0.11 ± 0.002; Atenolol: 0.09 ± 0.008; *p* = 0.03). In addition, there was an increase in lipid peroxidation and carbonyl protein levels in the atenolol group, TBARS (Control: 37.59 ± 3.01; Atenolol: 52.14 ± 1.09; *p* = 0.001), CP (Control: 6.66 ± 0.49; Atenolol: 15.17 ± 1.55; *p* = 0.0004).

**Fig. 4:**
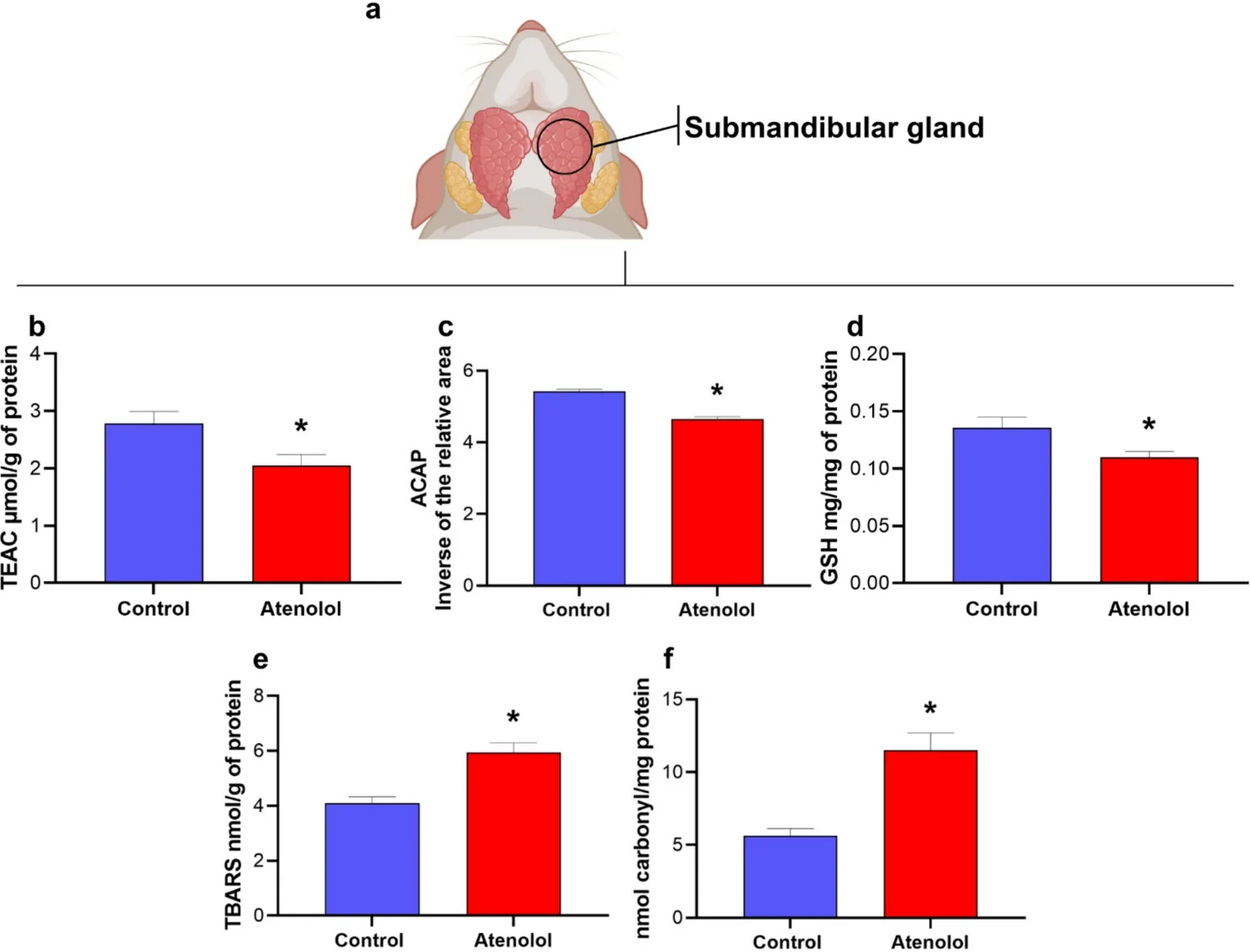
Effect of atenolol on the redox state of the submandibular gland. **A** Schematic representation of the anatomical location of the submandibular gland in rats. **B** TEAC—antioxidant capacity equivalent to Trolox; **C** ACAP—Antioxidant capacity against peroxyl radicals; **D** GSH—glutathione levels. Graphical representation of the analysis of pro-oxidant parameter data (**E**) TBARS—thiobarbituric acid reactive substances (**F**) CP – Carbonyl Proteins. Oxidative biochemistry expressed as mean ± SEM, Student’s t-test, * *p* < 0.05

In the submandibular gland (Fig. 4), a reduction in TEAC levels was observed (Control: 2.78 ± 0.20; Atenolol: 2.05 ± 0.19; *p* = 0.03), ACAP (Control: 5.42 ± 0.06; Atenolol: 4.65 ± 0.06; *p* < 0.0001) and GSH (Control: 0.13 ± 0.009; Atenolol: 0.11 ± 0.005; *p* = 0.03) in the group treated with atenolol, with a statistically significant difference. At the same time, TBARS and CP levels in the group treated with atenolol were increased compared to the control group. TBARS (Control: 4.10 ± 0.22; Atenolol: 5.94 ± 0.33; *p* = 0.001) CP (Control: 5.62 ± 0.49 nmol/mg; Atenolol: 11.50 ± 1.21; *p* = 0.0005).

**Fig. 5:**
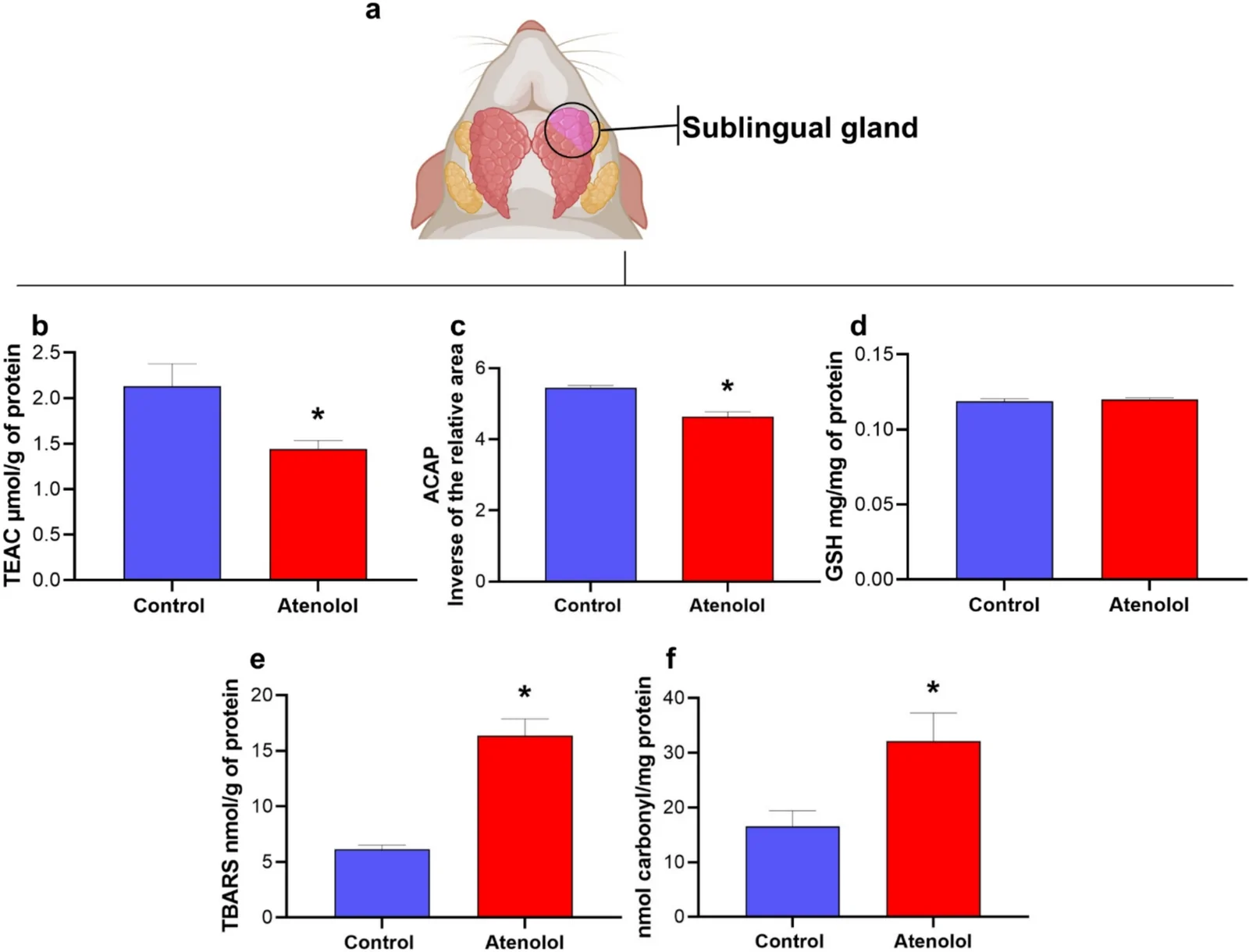
Effect of atenolol on the redox state of the sublingual gland. **A** Schematic representation of the anatomical location of the sublingual gland in rats. **B** TEAC—antioxidant capacity equivalent to Trolox; **C** ACAP—Antioxidant capacity against peroxyl radicals; **D** GSH—glutathione levels. Graphical representation of the analysis of pro-oxidant parameter data (**E**) TBARS—thiobarbituric acid reactive substances (**F**) CP – Carbonyl Proteins. Oxidative biochemistry expressed as mean ± SEM, Student’s t-test, * *p* < 0.05

In the sublingual gland (Fig. 5), the data revealed an antioxidant reduction in the analysis of TEAC (Control: 2.13 ± 0.24; Atenolol: 1.44 ± 0.09; *p* = 0.03), ACAP (Control: 5.44 ± 0.07; Atenolol: 4.63 ± 0.13; *p* = 0.0004) and GSH (Control: 0.11 ± 0.001; Atenolol: 0.12 ± 0.001; *p* = 0.55) in the atenolol group compared to the control. In addition, TBARS and CP levels were higher in the atenolol group (TBARS—Control: 6.13 ± 0.37; Atenolol: 16.35 ± 1.51; *p* = 0.0002; CP—Control: 16.57 ± 2.85; Atenolol: 32.14 ± 5.13; *p* = 0.02).

### The histopathology of the salivary glands was affected by atenolol administration

**Fig. 6:**
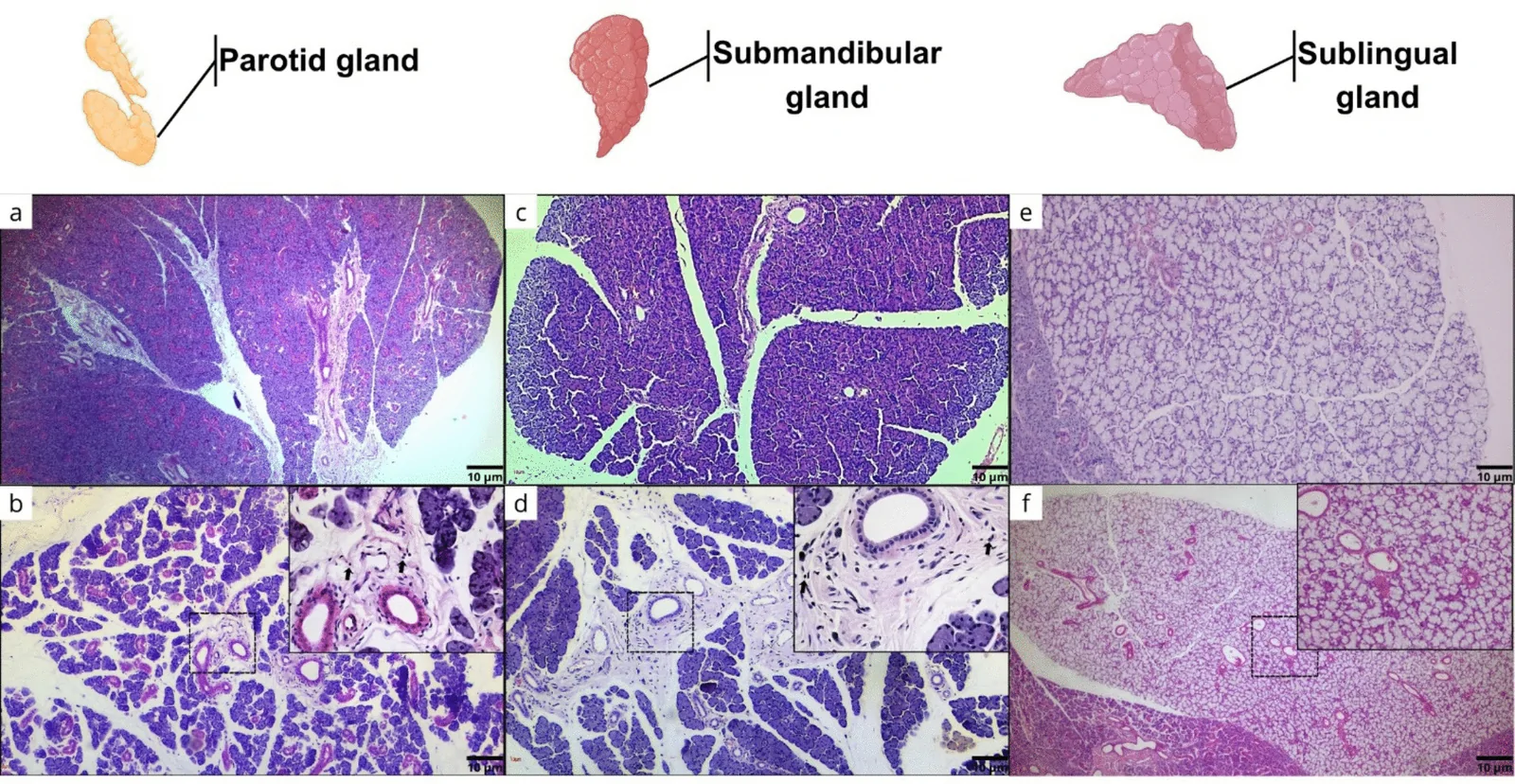
Histopathological changes evidenced in the parotid, submandibular, and sublingular glands exposed to atenolol. **A** Parotid gland of the control group with normal glandular parenchyma (H&E, 100x). **B** Parotid gland with acinar atrophy and presence of sparse lymphocytes in the glandular stroma in the group exposed to atenolol (H&E, 400x). **C** Submandibular gland of the control group with normal glandular parenchyma (H&E, 100x). **D** Submandibular gland of the group exposed to atenolol with mild acinar atrophy and presence of mast cells and sparse lymphocytes in the glandular stroma (H&E, 400x). **E** Sublingual gland of the control group with normal pattern (H&E, 100x). **F** Sublingual gland of the group exposed to atenolol with eosinophilic pattern (H&E 400x). Scale bar: 10 µm. Black arrows: in A, represents lymphocytes; in B represents mast cells

Based on comparative histopathological analysis (Fig. 6), when compared to the control group which presented normal glandular parenchyma, the parotid glands of the group exposed to atenolol showed morphological alterations such as acinar atrophy characterized by a significant reduction in the mean cross-sectional area of the acinar units, with the presence of sparse lymphocytes in the glandular stroma. Regarding the submandibular gland, the group exposed to atenolol presented mild acinar atrophy with the presence of mast cells and sparse lymphocytes in the glandular stroma. Furthermore, a pattern of eosinophilia was observed in the sublingual gland of the group exposed to atenolol, compared to the control group.

### Atenolol caused a reduction in the acinar area and stroma of the salivary glands

**Fig. 7:**
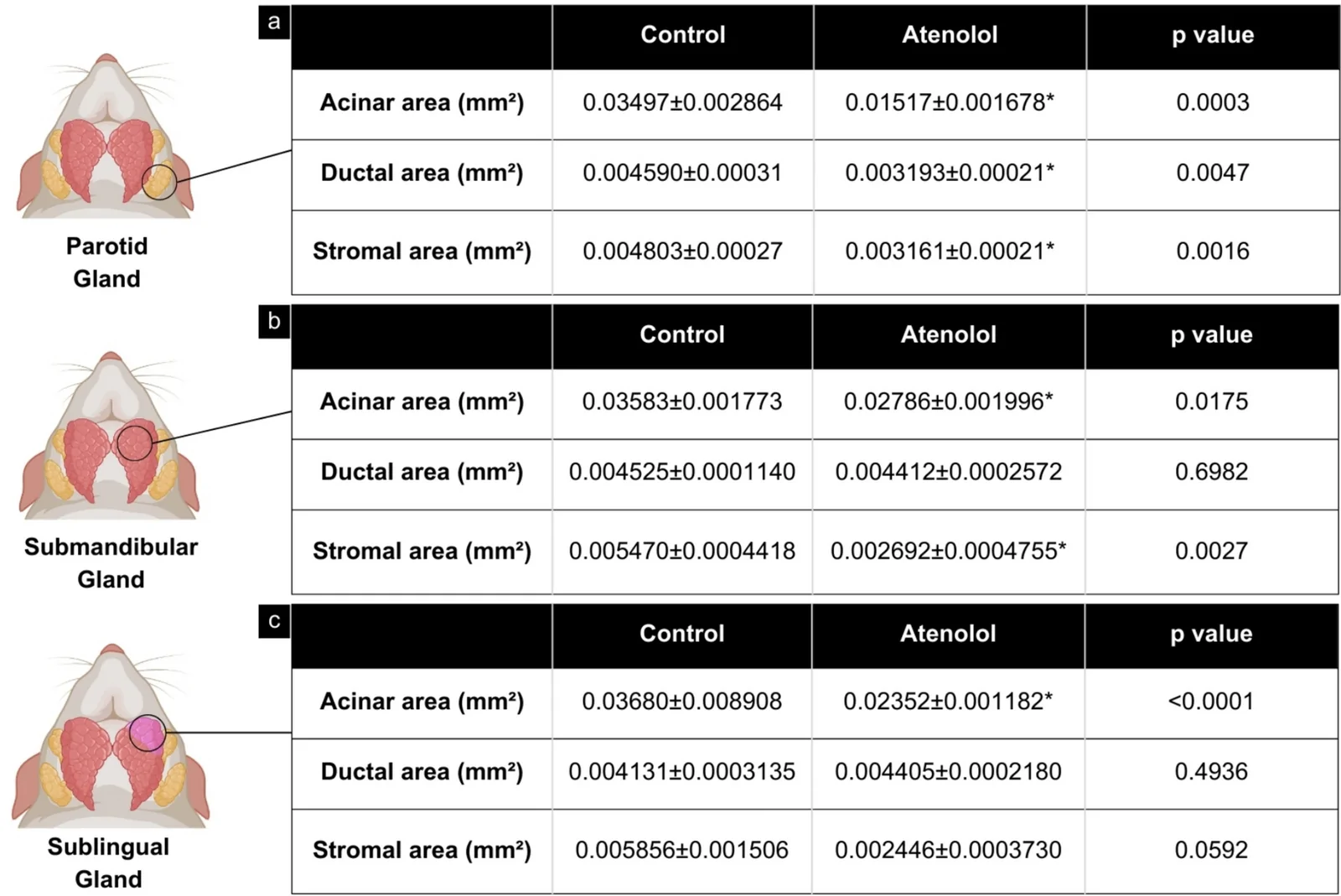
Raw morphometric data (acinar, ductal, and stromal area) of the salivary glands after 30 days of atenolol administration. **A** Parotid gland (**B**) Submandibular gland (**C**) Sublingual gland. Results are expressed as mean ± SEM. Student’s t-test, * *p* < 0.05

In the evaluation of the effects of atenolol on the histomorphometry of the salivary glands (Fig. 7), a reduction in the acinar area was observed in the three glands evaluated. In addition, in the evaluation of the ductal area, the parotid gland of the atenolol group showed a reduction compared to the control group, while the submandibular and sublingual glands showed no difference between the groups. Finally, in the measurement of the stromal area, the parotid and submandibular glands of the atenolol group showed a reduction compared to the control group, while the sublingual gland showed no difference between the groups.

### Atenolol affected collagen fibers in the parotid and submandibular glands

**Fig. 8:**
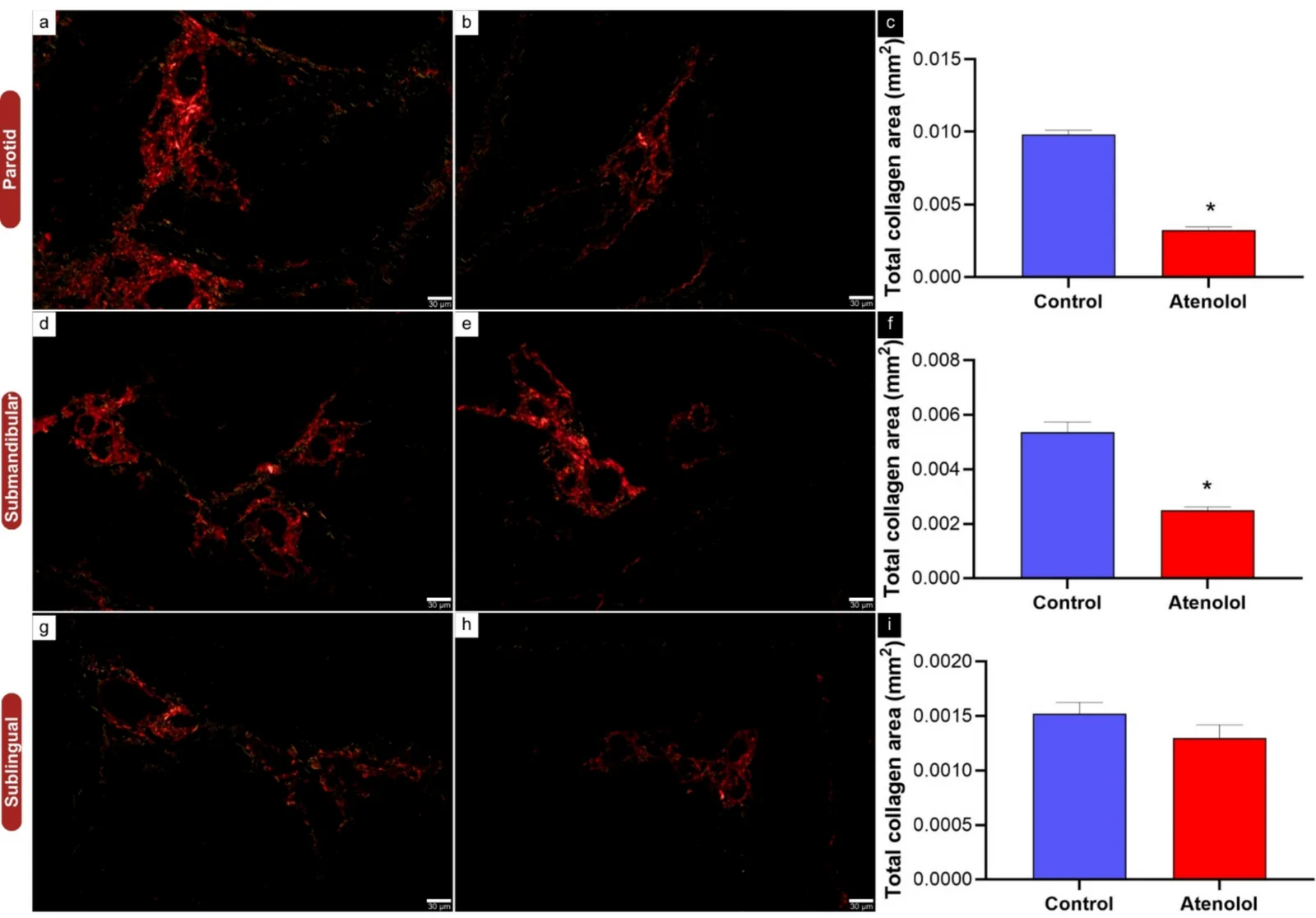
Picrosirius Red analysis to measure the total collagen area. Parotid gland (**A**, **B**, and **C**) (100x); submandibular gland (**D**, **E**, and **F**) (100x); sublingual gland (**G**, **H**, and **I**) (100x). Results are expressed as mean ± SEM. Student’s t-test, *p < 0.05. Scale bar: 30 µm

Histochemical analysis with picrosirius red (Fig. 8) revealed a significant reduction in the area of collagen fibers in the atenolol-treated group in the parotid glands (Control: 0.009 ± 0.0003; Atenolol: 0.003 ± 0.0002; *p* < 0.0001) and submandibular glands (Control: 0.005 ± 0.0003; Atenolol: 0.002 ± 0.0001; *p* < 0.0001). In the sublingual glands, no statistical difference was observed in the data (Control: 0.001 ± 0.0001; Atenolol: 0.001 ± 0.0001; *p* = 0.19).

### Atenolol caused an imbalance in the redox state of rat saliva after 30 days of exposure

**Fig. 9:**
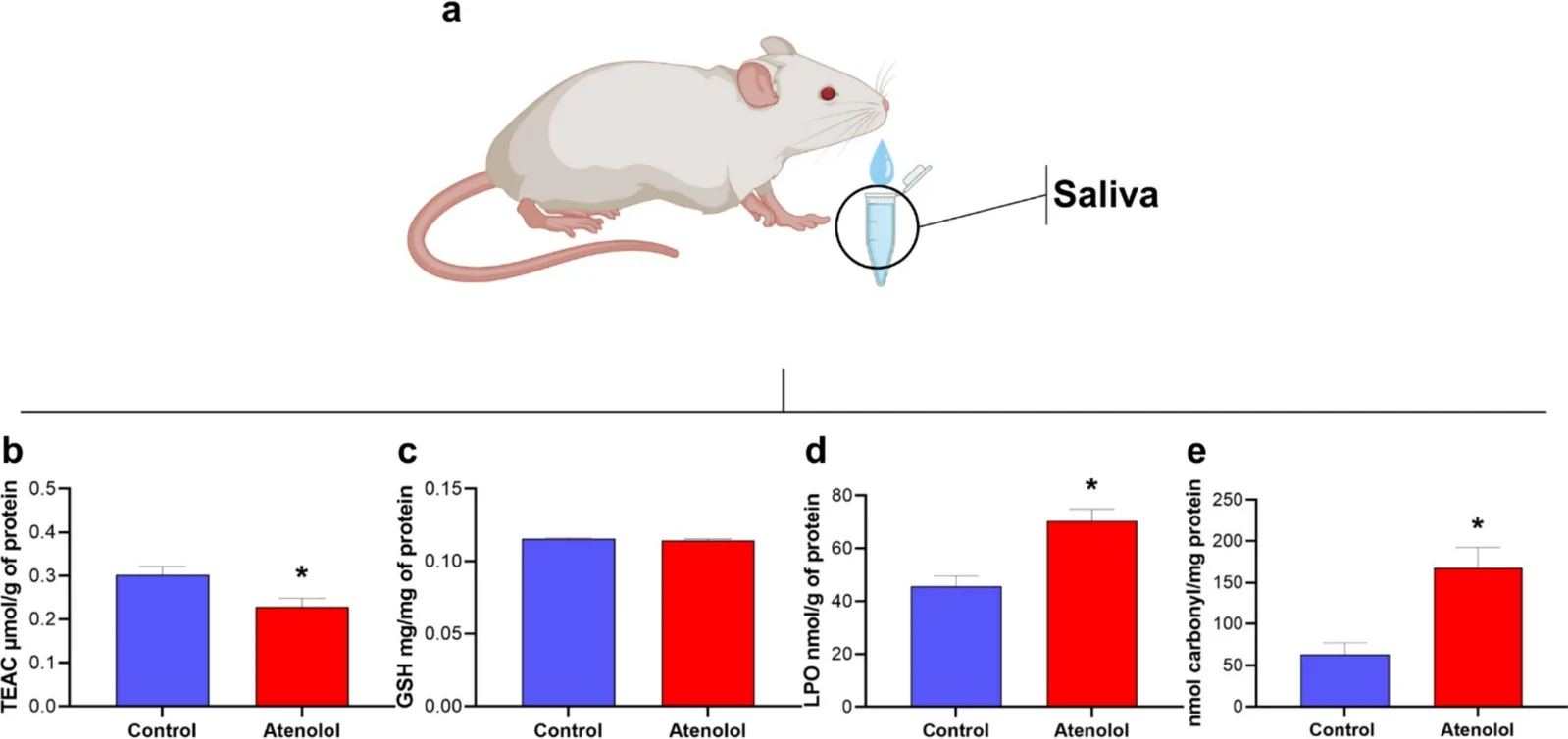
Effect of atenolol on redox state in saliva. **A** Schematic representation of the sample. Graphical representation of the analysis of pro-oxidant parameter data (**B**) TEAC—antioxidant capacity equivalent to Trolox; **C** GSH—glutathione levels; **D** LPO – Lipid Peroxidation; **E** CP – Carbonyl Proteins. Oxidative biochemistry expressed as mean ± SEM, Student’s t-test, * *p* < 0.05

When evaluating the oxidative biochemistry of saliva (Fig. 9), an imbalance in the redox state was observed, with decreased TEAC levels (Control: 0.30 ± 0.01; Atenolol: 0.22 ± 0.01; p = 0.02), while GSH levels showed no differences (Control: 0.11 ± 0.0006; Atenolol: 0.11 ± 0.0008; p = 0.28). On the other hand, pro-oxidant marker levels increased, such as LPO (Control: 45.74 ± 3.89; Atenolol: 70.36 ± 4.52; p = 0.002) and CP (Control: 63.20 ± 13.56; Atenolol: 167.8 ± 24.72; p = 0.006).

### Atenolol modulated protein and ion levels in saliva

**Fig. 10:**
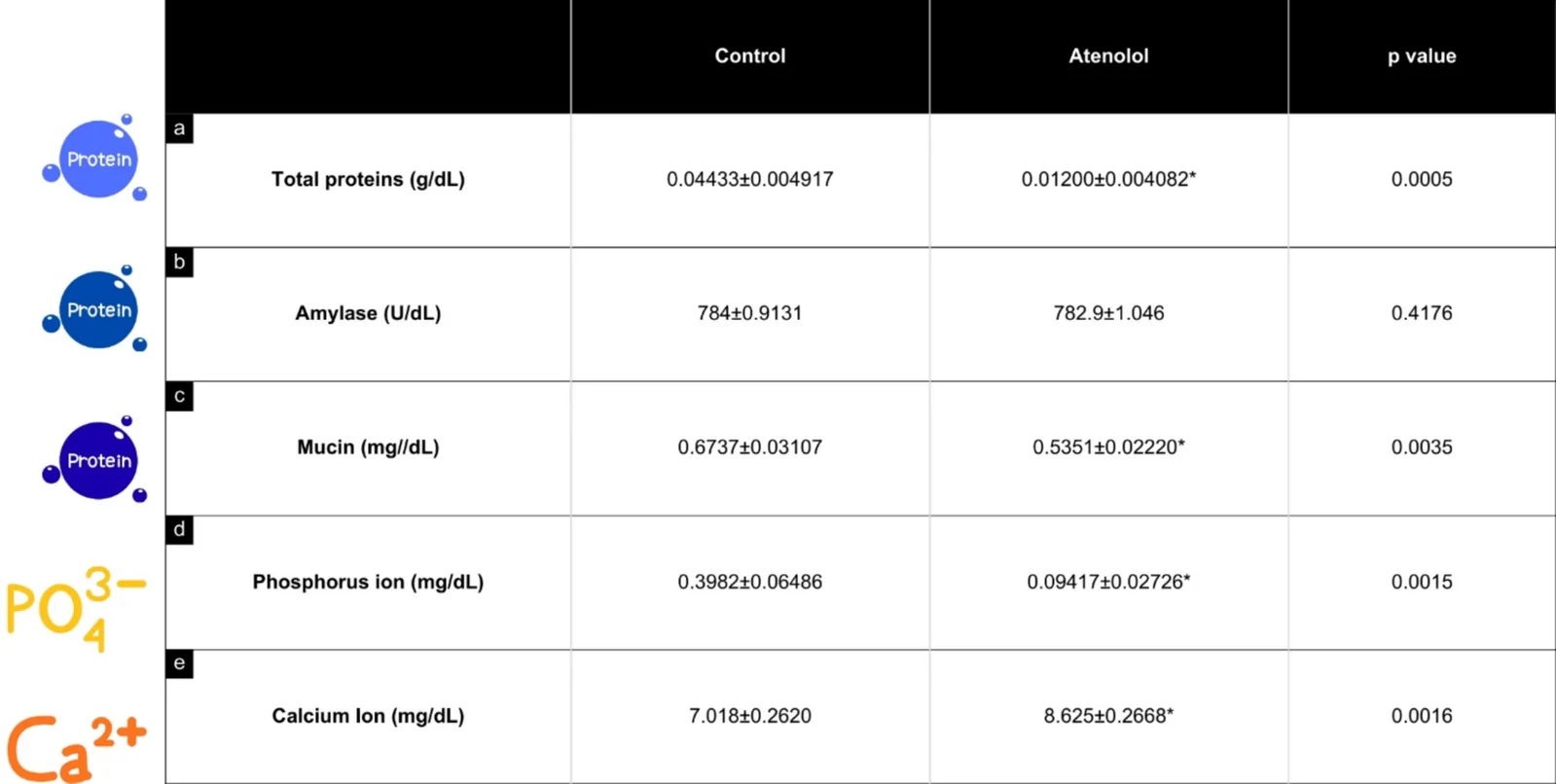
Salivary protein and ion assessment parameters. **A** Total proteins (**B**) Amylase (**C**) Mucin (**D**) Phosphorus (**E**) Calcium. Results are expressed as mean ± SEM. Student’s t-test, * *p* < 0.05

After 30 days of administration of atenolol at a dose of 20 mg/kg/day, the biochemical composition (Fig. 10) of saliva was affected, with a decrease in total protein and mucin levels, while amylase remained unchanged between groups. In ionic parameters, there was a decrease in phosphate ions and an increase in calcium ions.

## Discussion

Our results show, for the first time, that atenolol administration in rats is associated with oxidative alterations in all major salivary glands and saliva. This biochemical imbalance was accompanied by structural changes, including acinar atrophy, stromal reduction, and decreased collagen fibers, which were more pronounced in the parotid and submandibular glands. These findings suggest that sialochemical disturbances and glandular alterations observed after atenolol exposure are associated with increased oxidative stress in both tissue and saliva.

The integrity of these glands is essential for oral health, as they support saliva production and maintain functions such as lubrication, initial digestion, acid buffering, dental remineralization, and immune defense (Ferreira et al. 2021). Our findings suggest that the observed glandular alterations may be associated with changes in salivary flow and composition, potentially impairing its protective functions and thereby contributing to conditions such as xerostomia, dental caries, periodontal diseases, opportunistic infections, and swallowing disorders (Millsop et al. 2017).

Although atenolol is widely used in the treatment of hypertension (Zhang et al. 2004; Mengue et al. 2016), with recognized cardioprotective effects, including reduction of heart rate (Gómez et al. 2014). The dose used in this study (20 mg/kg/day) is described in the literature as safe and free of acute toxicity (Xie et al. 2002; Xu et al. 2004). Moreover, in humans, certain adverse effects associated with atenolol, including hypersensitivity responses such as Drug Reaction with Eosinophilia and Systemic Symptoms, typically emerge within a window of 2 to 8 weeks after treatment initiation. Although our study did not focus on systemic hypersensitivity markers, the observation of structural damage and oxidative stress within this 4-week timeframe aligns with a period of high susceptibility to drug-induced complications in clinical practice, reinforcing the translational importance of this experimental model (Mehmood et al. 2024; Silva et al. 2024). Previous studies show that atenolol can improve cardiovascular and immunological parameters (Gómez et al. 2014). However, growing evidence indicates that this drug is also capable of inducing oxidative imbalance in different biological models, including zebrafish (Adedara et al. 2024), mice (Sanchez-Roman et al. 2014) and humans (Deoghare and Kantharia 2017). These data suggest that atenolol can affect various tissues through different mechanisms, although its effects on salivary glands have not yet been investigated, reinforcing the relevance and originality of the present study.

To determine whether the changes associated with atenolol were systemic or tissue-specific, we evaluated peripheral blood alongside the salivary glands. Interestingly, no oxidative alterations were identified in plasma, as indicated by stable antioxidant parameters (TEAC and GSH) and nitric oxide metabolites. This contrast suggests a greater tissue-specific susceptibility of the salivary glands to atenolol, with a localized redox imbalance observed despite the maintenance of systemic redox homeostasis. Decreased levels of TEAC, ACAP, and GSH may reflect depletion of the tissue antioxidant defense system, possibly associated with increased production of reactive oxygen species in an attempt to maintain oxidative balance (Barbosa et al. 2010). In turn, increased TBARS and protein carbonylation may indicate the presence of oxidative alterations, as lipid peroxidation and protein oxidation are classical markers of oxidative stress, assessed by malondialdehyde formation and protein carbonyl content, respectively (Ayala et al. 2014; Dalle-Donne et al. 2006).

Furthermore, considering this increase in lipid peroxidation evidenced by the elevated TBARS levels in the atenolol-exposed group, it is relevant to discuss the possible role of ferroptosis in this context. Ferroptosis is considered a form of regulated cell death characterized by the accumulation of iron-dependent lipid hydroperoxides and the failure or reduction of antioxidant systems, particularly those associated with glutathione and glutathione peroxidase 4 (GPX4) activity (Dixon et al. 2012; Stockwell et al. 2017). Recent studies demonstrate that increased ROS, coupled with GSH depletion and intensified lipid peroxidation, resulted in an environment favorable to the activation of this pathway in different tissues (Jiang et al. 2021). Although ferroptosis has not yet been directly investigated in salivary glands, emerging evidence suggests that it may play an important role in oral conditions and inflammatory processes mediated by oxidative stress (Liu et al. 2020; Xiong et al. 2022). In this sense, according to our findings, such as the increase in TBARS and reduction in GSH in the salivary glands of the groups exposed to atenolol, it can be suggested that the mechanisms associated with ferroptosis may contribute to the tissue damage observed after exposure to this drug (Xiong et al. 2022). However, it is important to highlight those future investigations involving specific markers of this pathway, such as GPX4, ACSL4, and intracellular iron accumulation, are necessary to confirm this possibility and clarify its potential as a therapeutic target in alterations in salivary glands as well as in saliva.

This oxidative imbalance is potentially linked to atenolol’s ability to inhibit succinate dehydrogenase, an essential enzyme in the mitochondrial respiratory chain, potentially leading to an exacerbated production of reactive oxygen species in mitochondria (Seydi et al. 2020). Such a cascade could trigger the collapse of the mitochondrial membrane potential, compromising the integrity of the organelles and suggesting the activation of the intrinsic pathway of apoptosis mediated by cytochrome C (Seydi et al. 2020). Consequently, these events might promote the upregulation of inflammatory mediators, such as nuclear factor kappa B (NF-κB), in addition to the release of Damage-Associated Molecular Patterns (Seydi et al. 2020). The combination of these pathways likely contributes to the amplification of the tissue’s inflammatory response.

Our histopathological findings indicate that atenolol administration is associated with an inflammatory process in the salivary glands, as evidenced by the presence of cellular infiltrate composed of lymphocytes and mast cells, possibly related to the previously described local redox imbalance (Chamusca et al. 2012). In addition to these findings, morphometric and histochemical analyses were performed to further characterize glandular structural alterations. Morphometric analysis revealed acinar atrophy in all salivary glands evaluated. A reduction in ductal area was observed exclusively in the parotid gland, whereas stromal alterations were identified in the parotid and submandibular glands. Histochemical analysis demonstrated a decrease in collagen fibers in these glands, except for the sublingual gland, which did not show relevant changes in this parameter. Considering that the parotid and submandibular glands are primarily responsible for the production of salivary components (Chibly et al. 2022), structural alterations in these glands may be associated with changes in salivary flow and composition in the oral cavity (Talha and Swarnkar 2023).

It is important to highlight that acini constitute the main secretory units of the salivary glands, being responsible for the production of saliva, which is subsequently conducted to the oral cavity through the ductal system. The observed acinar atrophy can be explained by the mechanism of action of atenolol. As salivary gland development (Bloom et al. 1981) and salivary secretion is mediated by β-adrenergic receptors, prolonged blockade of these receptors by a beta-blocker can compromise secretory activity and favor atrophy of the glandular units, as described by Chen et al. (2020) and Wang et al. (2021). Thus, the identified structural alterations appear to result from the interaction between the pharmacological blockade of β receptors, the induced oxidative stress, and the subsequent tissue inflammatory response, contributing to the functional impairment of the salivary glands.

Considering the functional organization of tissues, structural changes tend to reflect functional impairments (Fernandes et al. 2015), which seems to be the case observed in this study. The cellular changes identified indicate deficits in salivary production and excretion, an essential factor for maintaining oral homeostasis (Farooq and Bugshan 2021). Functionally, these changes may be associated with xerostomia, characterized by a dry mouth sensation, since atenolol significantly reduced total protein and mucin levels, in addition to altering phosphorus and calcium ion concentrations, which are essential for salivary pH regulation (Talha and Swarnkar 2023). Previous studies involving the offspring of rats and children between 2 and 10 years of age also demonstrated a reduction in total protein levels in saliva, corroborating the findings of the present study (Al-Al–Sandook et al. 2008; Elias et al. 2008).

Functionally, these changes manifest as a selective modulation of the sialochemical profile, the reduction in total protein and mucin, without affecting amylase activity, highlights a divergence in cellular secretion pathways. Mucin secretion depends strictly on the β-receptor/cyclic AMP pathway (cAMP), which was directly targeted by atenolol. On the other hand, amylase activity may have been sustained by compensatory mechanisms, such as the calcium-mediated pathway via muscarinic (M3) and alpha-adrenergic receptors, which remain functional (Yoshida et al. 1967; Baum and Wellner 1999; Ryberg et al. 2008). This selective blockade not only alters qualitative saliva composition but may also induce secretory stasis and metabolic stress, further contributing to the redox imbalance (Proctor and Carpenter 2014; Proctor 2016).

Among the proteins present in saliva, mucin is vitally important for lubrication and maintenance of the oral cavity microbiota (Frenkel and Ribbeck 2015). The correlation between tissue damage and functional changes in saliva found in this study is interesting, as it is due to the presence of significant oxidative biochemical changes and morphometric changes in the salivary glands of the exposed group, which may be linked to a significant reduction in mucin production, mainly produced by the sublingual gland (Troxler et al. 1997) Changes in salivary quality and flow can significantly compromise oral health and quality of life, considering the essential role of saliva in digestive processes, protection against infectious agents, and lubrication of the oral mucosa (Dinu et al. 2025).

The distribution of beta-adrenergic receptors in the major salivary glands is not uniform, which results in different effects of atenolol on the salivary glands. The parotid gland has the highest density of the beta1 subtype, whose activation induces predominantly serous saliva, characterized by low volume and high concentrations of proteins and enzymes, whereas the submandibular and sublingual glands exhibit a more heterogeneous expression and are responsible for the secretion of mucous components via sympathetic signaling (Putney 1986; Ryberg et al. 2008; Proctor and Carpenter 2014).

Despite the robust results, this study presents some limitations that should be considered. The use of normotensive male animals may not fully reflect the toxicological capacity of atenolol under different physiological conditions. Furthermore, the absence of molecular analyses prevented the elucidation of the possible mechanisms involved in atenolol toxicity in salivary glands, as well as the identification of any compensatory mechanisms resulting from beta-receptor blockade. Additionally, it was not evaluated whether the observed damage pattern persists for longer periods (such as 60 and 90 days). Another relevant limitation concerns the possible effects of the anesthetic protocol on oxidative, metabolic, inflammatory, and molecular parameters. Studies indicate that anesthetic agents can increase ROS production, alter antioxidant systems, induce acute hyperglycemia, possibly mediated by alterations in insulin secretion and activation of α2-adrenergic receptors, and modulate inflammatory mediators such as TGF-β. Considering that hyperglycemia is associated with reduced salivary flow and salivary gland dysfunction, these effects could theoretically influence our assessed outcomes (Saha et al. 2005; Kadhim et al. 2025). Furthermore, evidence demonstrates that some anesthetics, including ketamine, can induce alterations in gene expression and protein transcription even after acute exposures, modulating pathways related to inflammation and cellular metabolism (Irwin et al. 2023; Upton et al. 2020). However, it is important to emphasize that all animals used in our study (both the control group and the group exposed to atenolol) underwent the same anesthetic protocol and the same cholinergic stimulus with pilocarpine, thus ensuring the standardization of these variables among the experimental groups. Therefore, although it is not possible to completely exclude the influence of these factors, it is suggested that they occurred similarly in all groups, thus not compromising the comparative interpretation of the results.

## Conclusion

Based on this preclinical study, atenolol may represent a potential risk factor for oral health, as its use was associated with sialochemical alterations and structural changes in salivary glands, accompanied by increased oxidative stress markers in both glandular tissue and saliva.

## Data Availability

All source data for this work (or generated in this study) are available upon reasonable request.
